# Genome-wide association studies for feed intake and efficiency in two laying periods of chickens

**DOI:** 10.1186/s12711-015-0161-1

**Published:** 2015-10-16

**Authors:** Jingwei Yuan, Kehua Wang, Guoqiang Yi, Meng Ma, Taocun Dou, Congjiao Sun, Lu-Jiang Qu, Manman Shen, Liang Qu, Ning Yang

**Affiliations:** National Engineering Laboratory for Animal Breeding and MOA Key Laboratory of Animal Genetics and Breeding, College of Animal Science and Technology, China Agricultural University, Beijing, 100193 People’s Republic of China; Jiangsu Institute of Poultry Science, Yangzhou, 225125 People’s Republic of China

## Abstract

**Background:**

Feed contributes to over 60 % of the total production costs in the poultry industry. Increasing feed costs prompt geneticists to include feed intake and efficiency as selection goals in breeding programs. In the present study, we used an F_2_ chicken population in a genome-wide association study (GWAS) to detect potential genetic variants and candidate genes associated with daily feed intake (FI) and feed efficiency, including residual feed intake (RFI) and feed conversion ratio (FCR).

**Methods:**

A total of 1534 F_2_ hens from a White Leghorn and Dongxiang reciprocal cross were phenotyped for feed intake and efficiency between 37 and 40 weeks (FI1, RFI1, and FCR1) and between 57 and 60 weeks (FI2, RFI2, and FCR2), and genotyped using the chicken 600 K single nucleotide polymorphism (SNP) genotyping array. Univariate, bivariate, and conditional genome-wide association studies (GWAS) were performed with GEMMA, a genome-wide efficient mixed model association algorithm. The statistical significance threshold for association was inferred by the simpleM method.

**Results:**

We identified eight genomic regions that each contained at least one genetic variant that showed a significant association with FI. Genomic regions on *Gallus gallus* (GGA) chromosome 4 coincided with known quantitative trait loci (QTL) that affect feed intake of layers. Of particular interest, eight SNPs on GGA1 in the region between 169.23 and 171.55 Mb were consistently associated with FI in both univariate and bivariate GWAS, which explained 3.72 and 2.57 % of the phenotypic variance of FI1 and FI2, respectively. The *CAB39L* gene can be considered as a promising candidate for FI1. For RFI, a haplotype block on GGA27 harbored a significant SNP associated with RFI2. The major allele of *rs315135692* was favorable for a lower RFI, with a phenotypic difference of 3.35 g/day between opposite homozygous genotypes. Strong signals on GGA1 were detected in the bivariate GWAS for FCR.

**Conclusions:**

The results demonstrated the polygenic nature of feed intake. GWAS identified novel variants and confirmed a QTL that was previously reported for feed intake in chickens. Genetic variants associated with feed efficiency may be used in genomic breeding programs to select more efficient layers.

**Electronic supplementary material:**

The online version of this article (doi:10.1186/s12711-015-0161-1) contains supplementary material, which is available to authorized users.

## Background

The long-term challenge for animal breeders is to improve the productivity of major livestock species to meet the growing demands for livestock products and minimize their impact on the environment and global natural resources [1]. An effective way to face this challenge is to improve feed intake and efficiency that play a decisive role in the economic benefit of livestock husbandry. Therefore, genetic improvement through the selection of animals with a greater ability to use feed could be one of the primary breeding goals. Generally, feed conversion ratio (FCR) and residual feed intake (RFI) were used to measure feed efficiency. FCR is widely used but not a suitable selection trait because of its complex correlations with growth and production traits [2]. RFI is defined as the difference between the observed and expected feed intake given a certain production, which is a sensitive and accurate measure for feed efficiency in chickens. Although traditional selection for RFI has made substantial genetic progress (see review [3]), it did not maintain the potential of current egg production of layers. However, selection based on genomic information could be a promising alternative [4].

In the previous decades, quantitative trait loci (QTL) for many traits in chicken have been studied. For feed intake (FI), RFI, and FCR, 37 QTL have been detected on various chicken chromosomes (http://www.animalgenome.org/cgi-bin/QTLdb/GG/index). However, these QTL mapping studies and candidate gene approaches are insufficient because of the low power of linkage analyses and bias in the detection of biologically plausible candidates for complex traits [5, 6].

Compared to previous studies, genome-wide association studies (GWAS) can involve larger genomic regions and detect smaller associated chromosomal regions, and provide more precise estimates of the size and direction of the effects of alleles at the associated loci [7]. Recently, GWAS have revealed many important findings associated with production traits, disease resistance, and morphological characteristics in chickens [8]. However, to date, no GWAS has been performed to evaluate the genetic architecture of feed intake and efficiency in chickens. In addition, the availability of the chicken 600 K high-density single nucleotide polymorphism (SNP) chip permits interrogation of the entire chicken genome at resolutions that were previously unattainable, making the genetic analysis more powerful. The purpose of our study was to identify genetic variants and candidate genes for feed intake and efficiency in chicken using the genome-wide association approach in an F_2_ resource population genotyped with the chicken 600 K SNP array.

## Methods

### Ethical statement

This study was conducted in accordance with the Guidelines for Experimental Animals established by the Animal Welfare Committee of the Chinese Agricultural University.

### Birds and phenotypes

An F_2_ resource population was produced by reciprocal crossing since 2011 between White Leghorn (WL) chicken that originated from the Shanghai Poultry Breeding Co., Ltd, with selection on egg production and quality, and Dongxiang Blue-shelled (DBS) chicken, an indigenous breed introduced from the Jiangxi province, China, with selection on egg production, eggshell color, and egg quality since 1998 at the research farm in the Jiangsu Institute of Poultry Science. WL is a global commercial layer breed that is recognized for its high egg production efficiency, whereas DBS is recognized for its blue eggshell color and meat quality. The two parental lines differed in various characters including morphological, physiological, and production traits. Reciprocal mating between unrelated individuals, i.e., 6 WL (♂) × 133 DBS (♀) and 6 DBS (♂) × 80 WL (♀), was used to produce the F_1_ generation. Then, 25 F_1_ males and 406 F_1_ females from WL (♂) × DBS (♀) and 24 F_1_ males and 233 F_1_ females from DBS (♂) × WL (♀) were randomly selected to produce the F_2_ generation, yielding 1856 males and 1893 females from 590 full-sib families in a single hatch as described previously [9].

A total of 2447 individuals were included in a 3-generation pedigree, of which 1534 hens had records for feed intake and efficiency during two separate laying periods, i.e. between 37 and 40 weeks and between 57 and 60 weeks of age. The phenotypes and production performances of the hens were collected as described previously [9]. Regarding how individual feed consumption was recorded: each hen had its own metal feed trough with mash feed, which was manually added. The total weight of each trough was measured, then two to three days later, the remaining weight was recorded and individual FI during this interval was calculated [9]. This process was repeated for 28 days during the testing period. The total feed intake for each hen was calculated by summing the feed consumption in each 2- or 3-day interval, and then used to calculate the daily FI of the laying period. RFI was calculated as the difference between actual FI and expected FI. A previously reported formula [10] was used to calculate the expected FI:

$${\text{E(FI) = b}}_{ 0} {\text{ + b}}_{ 1} {\text{MBW + b}}_{ 2} {\text{EM + b}}_{ 3} {\text{BWG,}}$$

where E(FI) is expected daily feed intake; MBW is metabolic body weight (BW raised to the power of 0.75), EM is daily egg mass (adjusted for abnormal eggs); BWG is daily body weight gain; b_0_, b_1_, b_2_, and b_3_ are partial regression coefficients.

### Genotyping, imputation, and quality control

Genomic DNA was extracted from blood samples using the phenol–chloroform method. The eligible DNA was quantified, and then genotyped using the 600 K Affymetrix Axiom high-density array. The SNP chip contained 580,961 SNPs across 28 autosomes, two linkage groups (LGE64 and LGE22C19W28_E50C23), and two sex chromosomes [11]. The Axiom™ GT1 algorithm was used for genotype calling of the 580,961 SNPs from the 1534 samples. The quality control of genotype data was performed with Affymetrix Power Tools (APT) developed by Affymetrix (http://affymetrix.com/): poor quality markers (for which genotyping failed in more than 5 % of the samples) and poor quality DNA samples (for which genotyping failed for more than 3 % of the markers) were removed. The SNP data quality was then controlled by PLINK software [12] to exclude from further analysis SNPs with a minor allele frequency less than 0.01 and those that deviated from the Hardy–Weinberg equilibrium (HWE) (*P* value < 1e−6). Finally, 435,867 autosomal SNPs and 1512 samples passed the quality control. To perform association analysis with GEMMA and infer the independent tests with SimpleM, and to increase the power of the GWAS analysis, genotype imputation [13] was performed. Missing genotypes were imputed based on information from pedigree and remaining SNP genotypes for the F_2_ population, as implemented in the Beagle Version 4 program with R-squared >0.3 [14]. Quality control of the imputed data was performed using PLINK software, which excluded SNPs that deviated from HWE. Finally, 435,243 SNPs and 1512 samples remained for the GWAS.

### Statistical analyses

All autosomal SNPs were pruned using the indep-pairwise option in PLINK, with a window size of 25 SNPs, a step of five SNPs, and a r^2^ threshold of 0.2; this resulted in 22,343 independent SNPs. Pairwise identity-by-state (IBS) distances were calculated between all individuals using the independent SNPs, and then multidimensional scaling (MDS) components were obtained using the MDS-plot option based on the IBS matrix. All options and parameters are similar to those previously used [15] and as implemented in PLINK software.

To account for population structure, a stratification approach was used in a GWAS using a mixed model [16] that included fixed effects (overall mean and covariates) and random effects (SNP effect, individual effect and residual errors), as implemented in GEMMA software [17]. Testing was done using a Wald test against the null hypothesis of g = 0. In the current study, all 1534 genotyped F_2_ birds were hatched from a single batch and raised in the same individual cages, therefore, only the first principal component was used as a covariate to account for population structure in the analysis. First, an association test using the univariate linear mixed model (univariate GWAS) was performed for each trait. The statistical model was as follows:

$${\mathbf{y}} = {\mathbf{W}\varvec{\upalpha}} + {\mathbf{x}}{\upbeta} \, + {\mathbf{u}} + {\varvec{\upvarepsilon}},$$

where **y** is the vector of trait values for all individuals; **W** is a matrix of covariates (fixed effects that contains the first MDS component and a column of 1s); **α** is a vector of the corresponding coefficients including the intercept; **x** is a vector of genotypes of a marker; β is the effect size of the marker; **u** is a vector of random individual effects; **ε** is a vector of errors.

To further characterize candidate regions that affect traits, we performed linkage disequilibrium (LD) analysis for the chromosomal regions with multiple clustered significant SNPs using the solid spin algorithm implemented in Haploview version 4.2 software [18]. Given the high genetic correlation for each trait between the two laying periods, we performed an association study using a bivariate linear mixed model (bivariate GWAS) [19], implemented in GEMMA software to detect the genes that were involved in both traits. Finally, a conditional analysis, including the most significant SNPs of a genome-wide scan as covariates into the model in a stepwise manner, was performed to identify secondary association signals [20].

Since Bonferroni correction is overly conservative due to the high LD in genetic data, it may produce false negative results [21]. Therefore, we calculated the number of effectively independent tests based on the SimpleM method [22] implemented in R software [23]. It includes three steps: (1) using the *cor* () function in R to derive the composite LD (CLD) correlation matrix from the SNP dataset; (2) using the R function *eigen* () to calculate the eigenvalues; and (3) inferring the effective number of independent tests through principal component analysis (PCA), which amounted to 59,286 independent tests. Then, the threshold *P*-value of the 5 % genome-wide significance was adjusted to 8.43 × 10^−7^ (0.05/59,286), and 1.69 × 10^−5^ (1.00/59,286) for suggestive significance.

In addition, SNP-based genetic correlations between traits recorded during the two laying periods, and phenotypic variances explained by the significantly associated SNPs were calculated using GCTA software [24, 25]. The annotated genes that were closest to significant SNPs were identified using Ensembl and NCBI annotation of the *Gallus gallus* 4.0 genome version.

## Results

Descriptive statistics for FI, RFI, and FCR in the two laying periods, and genetic correlations for FI, RFI, and FCR between the two laying periods are in Table 1. It should be noted that the multivariate linear model was fitted with all available hens (1856 and 1802 for each period) to estimate RFI, which may enhance the R-square of the model, compared to the model that was fitted with 1534 genotyped hens to estimate RFI, and hence obtained more accurate RFI values (see Additional file 1: Table S1). Therefore, we used the RFI value that was estimated from the model that considered the largest number of hens to calculate the mean RFI of the 1534 genotyped hens, which resulted in a non-zero mean RFI. Traits that did not follow a normal distribution were transformed using the Box-Cox or Johnson transformation implemented in R software [23].

**Table 1:** Descriptive statistics and genetic correlations (r_g_) between two laying periods for feed intake and efficiency

Traits^a^	Mean	SD	CV (%)	Min	Max	^b^R_g_
FI1 (g/day)	91.57	7.94	8.67	56.14	106.36	0.78 (0.09)
FI2 (g/day)	105.12	7.99	7.60	53.86	125.11	
RFI1 (g/day)	0.03	6.58	21,933	−24.44	23.73	0.76 (0.09)
RFI2 (g/day)	0.27	7.11	2633	−32.00	23.16	
FCR1 (g:g)	2.61	0.72	27.58	1.72	10.31	0.94 (0.07)
FCR2 (g:g)	3.61	3.08	85.31	1.83	61.96	

n = 1534

^a^FI1, RFI1 and FCR1, and FI2, RFI2 and FCR2 represent daily feed intake, residual feed intake and feed conversion ratio in laying period between 37 and 40 weeks and between 57 and 60 weeks, respectively

^b^r_g_ = genetic correlation; standard errors of estimates are in parentheses

### Feed intake

#### Univariate and bivariate genome-wide association studies

**Fig. 1:**
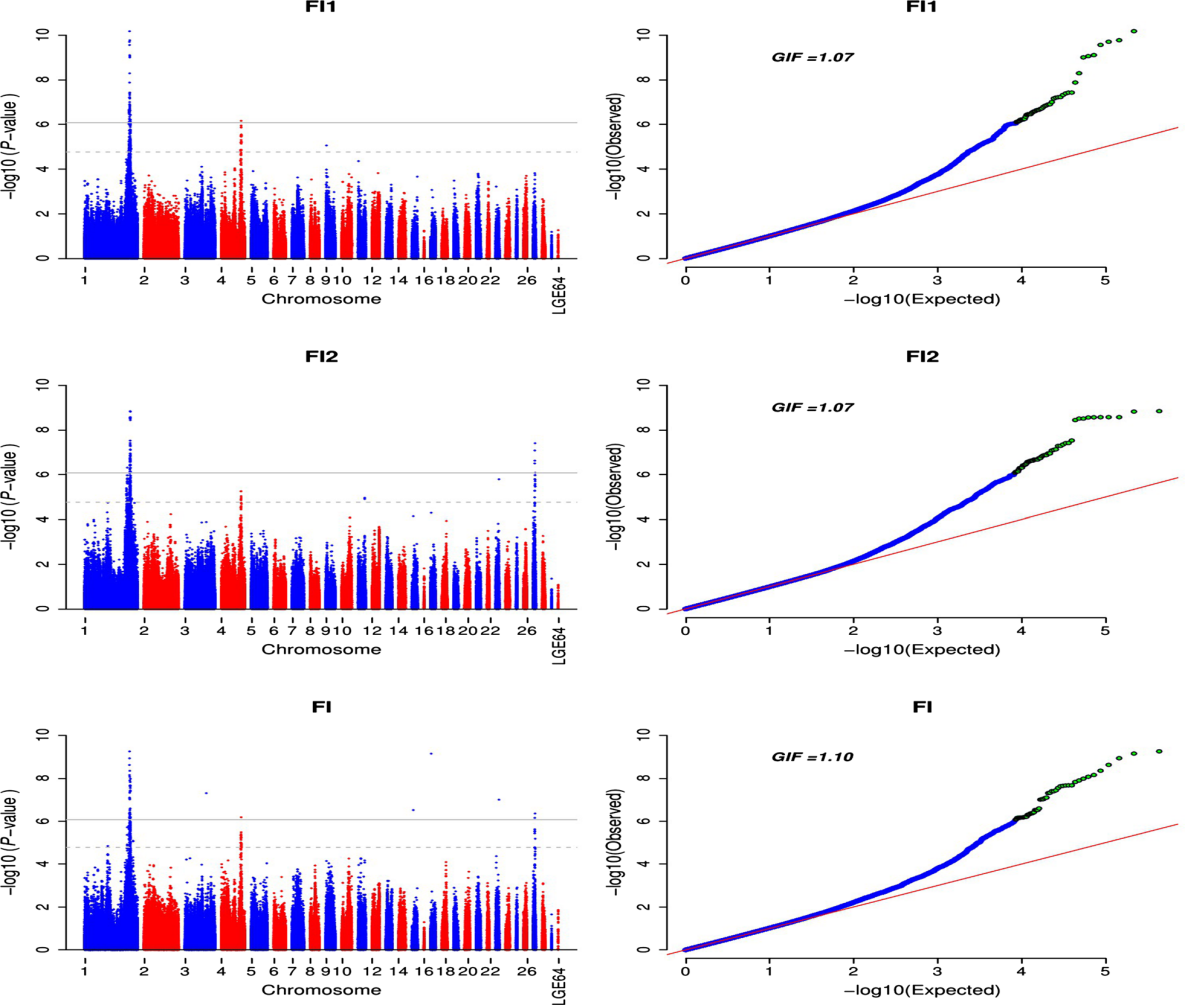
Univariate and bivariate genome-wide association studies for daily feed intake. *FI1* and *FI2* represent daily feed intake in laying periods between 37 and 40 weeks and between 57 and 60 weeks, respectively; *FI* represents daily feed intake in the bivariate analysis; *LGE64* linkage group LGE64, *GIF* genomic inflation factor; the *horizontal gray* and *gray dashed lines* indicate the whole-genome significance (*P* value = 8.43e−7) and genome-wise suggestive significance thresholds (*P* value = 1.69e−5), respectively

**Fig. 2:**
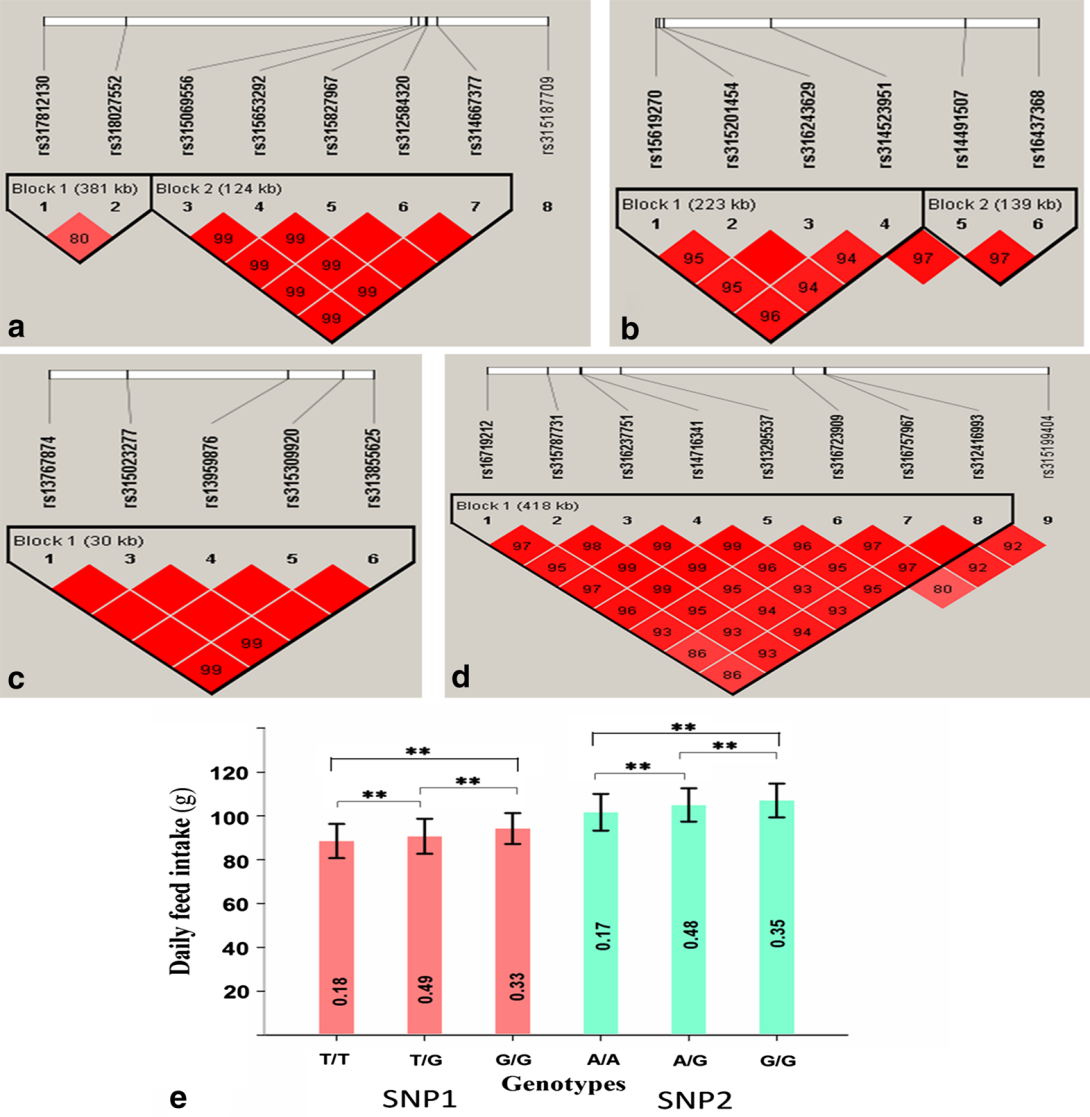
Significant regions associated with daily feed intake. **a** Linkage disequilibrium plot for the eight SNPs on GGA1 showing significant association with daily feed intake in univariate and bivariate GWAS. *Block 2* that contains the most significant SNP, i.e. *rs315069556* for daily feed intake in the laying period between 57 and 60 weeks, is in very high linkage disequilibrium (r^2^ > 0.99) with surrounding SNPs. **b** Linkage disequilibrium plot for the SNPs on GGA4 showing significant association with daily feed intake after conditional analysis in the laying period between 37 and 40 weeks. **c**, **d** Linkage disequilibrium plots for the SNPs on GGA1 and GGA27 showing significant association with daily feed intake after conditional analysis in the laying period between 57 and 60 weeks. *Solid lines* mark the identified blocks. **e** Genotype effect plot of two leading SNPs for daily feed intake in the first (between 37 and 40 weeks) and second laying periods (between 57 and 60 weeks), respectively. **(*P* < 0.01) indicates significant differences among groups; decimals inside the *bars* of **e** indicate genotype frequency; SNP1 = *rs14916642*; SNP2 = *rs315069556*

The Manhattan and Q–Q plot of the univariate and bivariate GWAS for daily feed intake are in Fig. 1. In the univariate analysis, 50 significant SNPs were associated with FI1. Among these 50 SNPs, 49 were located on *G. gallus* (GGA) chromosome 1 between 166.0 and 171.5 Mb, and one was located at 75.7 Mb on GGA4. For FI2, 47 significant SNPs were located on GGA1 between 159.5 and 171.7 Mb, and five significant SNPs with a low minor allele frequency (MAF < 0.1) were found on GGA27 between 30.9 and 37.8 Mb. The bivariate GWAS analysis resulted in association signals on the same chromosomes as with two univariate analyses, i.e. GGA1, GGA4, and GGA27. In addition, four other SNPs on GGA3, 15, 17, and 23 were significantly associated with FI in the bivariate analysis. Interestingly, a region on GGA1 between 169.23 and 171.55 Mb that contained eight SNPs was found to be associated with FI in both the univariate and bivariate analyses (Table 2). LD analysis showed that these multiple significant SNPs were located in two haplotype blocks that spanned 381 and 124 kb, respectively, of which the most significant SNP for FI2, i.e. *rs315069556*, was in block 2 (Fig. 2). Ideally, the genomic control inflation factors should be equal to 1.00. In the present study, genomic control inflation factors were slightly greater than 1 (λ = 1.07, 1.07, and 1.10 for each analysis, respectively), which reflected low population stratification.

**Table 2:** SNPs significantly associated with daily feed intake detected in both univariate and bivariate GWAS analyses

SNP	GGA^a^	Position	*P* value^b^	MAF^c^	β^d^		Candidate/nearest gene	Location (kb)^e^
					FI1	FI2		
rs317812130	1	169,231,399	9.22e−8	0.37 (A/G)	−0.26	−0.25	RNASEH2B	Intron 9
rs318027552	1	169,613,334	1.86e−7	0.34 (A/G)	−0.25	−0.24	ATP7B	D 3.41
rs315069556	1	170,920,135	2.60e−7	0.41 (A/G)	−0.24	−0.28	UFM1	U 38.06
rs315653292	1	170,954,280	2.11e−7	0.42 (G/A)	−0.24	−0.28	ENSGALT00000042093	U 63.24
rs315827967	1	170,989,520	2.11e−7	0.42 (C/A)	−0.24	−0.28	ENSGALT00000042093	U 98.48
rs312584320	1	170,993,003	2.11e−7	0.42 (C/T)	−0.24	−0.28	ENSGALT00000042093	U 101.96
rs314667377	1	171,044,869	2.45e−7	0.41 (A/C)	−0.24	−0.28	TRPC4	U 57.64
rs315187709	1	171,548,178	1.29e−7	0.40 (A/G)	−0.23	−0.27	FAM48A	U 26.65

^a^Chicken chromosome

^b^ *P* values are obtained from averages across the univariate and bivariate GWAS analyses

^c^Allele frequency of the first listed marker

^4^Effect of allele substitution obtained from averages across the univariate and bivariate GWAS analyses; FI1 and FI2 represent daily feed intake in laying period between 37 and 40 weeks and between 57 and 60 weeks, respectively

^e^U and D indicate that the SNP is upstream and downstream of a gene, respectively

#### Conditional analysis

We performed conditional analysis by fitting the most significant SNP in the model to test for additional associations. The resulting Manhattan and Q–Q plots are in Additional file 2: Figure S1. After conditional analysis, the previously significant SNPs for FI1 on GGA1 disappeared, whereas five SNPs on GGA4 between 75.5 and 76.2 Mb, which were in close proximity to the significant SNP that was detected in the univariate analysis, surpassed the genome-wide significance threshold. These five SNPs were clustered in two blocks by linkage analysis (Fig. 2), and they all had a low MAF (<0.1), thus, we chose SNP *rs314523951* previously detected in the univariate analysis as the leading SNP in the following analyses. Interestingly, a region on GGA1 that corresponded to a 30 kb haplotype block between 159.51 and 159.54 Mb included six SNPs (Fig. 2) for FI2 after conditional analysis, which suggested that this region harbors another QTL that affects FI2. Nine SNPs on GGA27 between 3.08 and 3.50 Mb were also significantly associated with FI2, of which eight were in a 418 kb long LD block (Fig. 2). After conditional analysis, genomic control inflation factors decreased to 1.02 for FI1 and 1.03 for FI2, respectively. Moreover, no significant association was found on GGA1 after conditional analysis of the results of the bivariate GWAS, and the genomic control inflation factor was reduced to 1.06.

#### SNP effects

For FI1, the phenotypic variance explained by all significant SNPs considered together was equal to 12.12 % [standard error (SE) = 0.05]. Two leading SNPs (*rs14916642* on GGA1 and *rs314523951* on GGA4) accounted for 5.44 and 1.93 % of the phenotypic variance, respectively (Table 3). For FI2, the phenotypic variance explained by all significant SNPs was equal to 10.99 % (SE = 0.05). Three leading SNPs (*rs315069556* on GGA1, *rs316723909* on GGA27, and *rs315557710* on GGA1) explained 4.41, 2.37, and 2.69 % of the phenotypic variance, respectively (Table 3). The genotype effects of the two most significant SNPs for FI1 and FI2 are in Fig. 2, which indicates that FI differs significantly between the three genotypes. The level of feed consumption of hens with genotype *TT* at SNP *rs14916642* on GGA1 was lowest during the first laying period, while hens with genotype *AA* at SNP *rs315069556* ate significantly less than those with genotypes *AG* and *GG* during the second laying period. In the bivariate analysis, the eight SNPs that were most likely associated with FI during the two laying periods explained 3.72 and 2.57 % of the phenotypic variance for FI1 and FI2, respectively.

**Table 3:** Leading SNPs associated with feed intake and efficiency in two laying periods

Traits^a^	SNP	GGA^b^	Position	*P* value^c^	MAF^d^	β^e^	CPV % (se)^f^	Candidate/nearest gene	Location (kb)^g^
FI1	rs14916642	1	168,476,105	1.40e−11*	0.42 (T/G)	−0.32	5.44 (0.07)	CAB39L	Intron 4
	rs314523951	4	75,701,319	4.01e−7*	0.22 (T/C)	0.23	1.93 (0.03)	LDB2	U 55.53
FI2	rs315069556	1	170,920,135	1.44e−9*	0.41 (A/G)	−0.29	4.41 (0.06)	UFM1	U 38.06
	rs316723909	27	3,464,323	3.91e−8*	0.09 (G/A)	0.38	2.37 (0.03)	GIP	U 3.78
	rs315557710	1	159,514,826	2.35e−7*	0.45 (T/A)	−0.22	2.69 (0.04)	LOC427010	D 1510.07
RFI1	rs317358229	2	10,015,354	1.52e−5	0.31 (C/T)	−5.57	1.62 (0.02)	DIP2C	U 15.58
	rs314206946	5	16,019,472	3.42e−6	0.33 (C/T)	5.83	1.82 (0.03)	LOC423119	intron 9
RFI2	rs315135692	27	3,247,383	6.89e−7*	0.30 (A/G)	9.43	1.77 (0.03)	LOC429785	exon 1
FCR1	rs13870350	1	56,004,454	6.33e−6	0.34 (A/G)	0.22	1.98 (0.03)	TBXAS1	intron 6
	rs317101341	7	16,787,525	2.59e−6	0.05 (A/C)	0.45	1.69 (0.02)	CDCA7	D 42.52
FCR2	rs312620976	11	642,639	1.86e−6	0.21 (C/T)	0.24	1.85 (0.03)	CETP	D 16.04

^a^FI1, RFI1 and FCR1, and FI2, RFI2 and FCR2 represent daily feed intake, residual feed intake and feed conversion ratio in laying periods between 37 and 40 weeks and between 57 and 60 weeks

^b^Chicken chromosome

^c^* Indicates that the SNP *P* value reaches a genome-wise significance

^d^Allele frequency of the first listed marker

^e^Effect of allele substitution

^f^Contribution to phenotypic variance and standard error

^g^U and D indicate that the SNP is upstream and downstream of a gene, respectively

### Residual feed intake

**Fig. 3:**
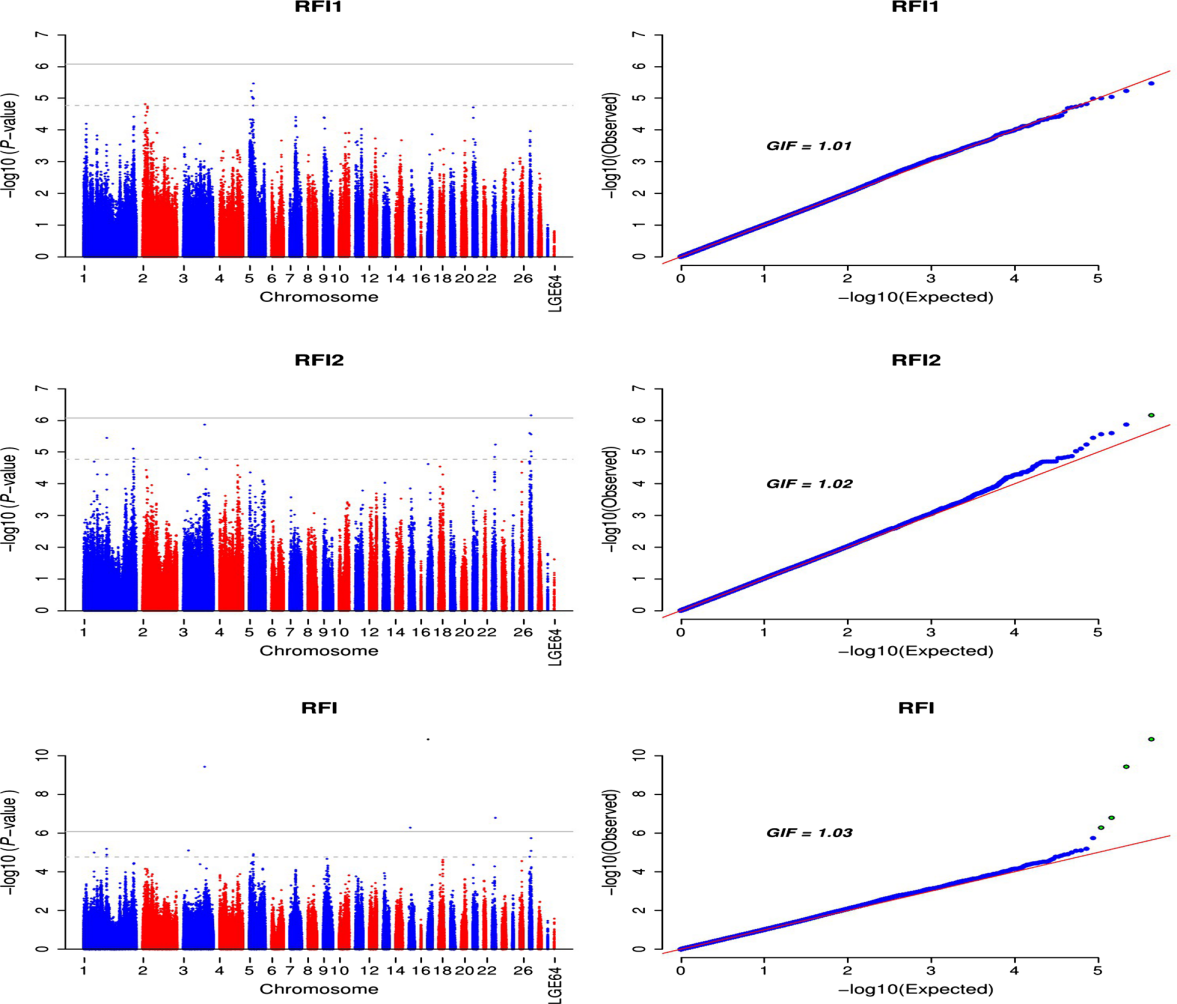
Manhattan and Q–Q plots of genome-wide association *P* values for residual feed intake. *RFI1* and *RFI2* represent residual feed intake in laying periods between 37 and 40 weeks and between 57 and 60 weeks, respectively; *RFI* represents residual feed intake in the bivariate analysis; *LGE64* linkage group LGE64, *GIF* genomic inflation factor. The *horizontal gray* and *gray dashed lines* indicate the whole-genome significance (*P* value = 8.43e−7) and genome-wise suggestive significance thresholds (*P* value = 1.69e−5), respectively

In the present study, 20 SNPs, including seven SNPs associated with RFI1 and 13 SNPs associated with RFI2, reached the genome-wise suggestive significance level (*P* value < 1.69e−5) in the univariate GWAS analysis (Fig. 3; Additional file 3: Table S4). Among the seven SNPs associated with RFI1, six were located on GGA5 and one on GGA2. Together, these SNPs explained 4.07 % (SE = 0.03) of the phenotypic variance. The most significant SNP (*rs314206946*, *P*-value = 3.24e−6, allele substitution effect equal to 5.83 units of RFI) was located on GGA5 in an intron of a novel gene *LOC423119* (*fatty acid desaturase 1*-*like*) and 21.6 kb upstream of the *CPT1A* (*carnitine palmitoyltransferase 1A*) gene (Table 3). The minor allele of SNPs on GGA5 had positive effects (β > 0) on RFI1, whereas the minor allele of SNPs on GGA2 had negative effects. SNPs associated with RFI2 were detected on GGA1, 3, 23, and 27, and together explained 10.22 % (SE = 0.04) of the phenotypic variance. The synonymous coding SNP (*rs315135692*, *P*-value = 6.89e−7) that was situated in exon 1 of the *LOC429785(G protein*-*activated inward rectifier potassium channel 1*-*like*) gene on GGA27 satisfied our criteria for genome-wide significance (Table 3). The minor allele of this SNP had a positive effect on RFI2 and contributed to 1.77 % (SE = 0.03) of the phenotypic variance of RFI2. Moreover, the mean RFI values for each of the three genotypes at SNP *rs315135692* differed significantly (*P*-value < 0.01) and the chicken with genotype *GG* had a lower RFI value (-0.58 ± 7.81) than those with genotypes *AG* (0.75 ± 7.14) and *AA* (2.77 ± 6.79), which indicates that they are the most efficient during the second laying period. Interestingly, SNP *rs14290671* on GGA23 is a missense variant located in the *GNL2* (*guanine nucleotide binding protein*-*like 2 (nucleolar)*) gene, which causes a transformation from phenylalanine to cysteine, and the SIFT [26] score is equal to 0.06, which is close to the damaging score 0.05.

In the bivariate analysis, four isolated hits, which were identical to SNPs detected for FI, were significantly associated with RFI (Fig. 3; Table 4). Positional candidate genes of four SNPs were identified as: *HTR1B* (*5*-*hydroxytryptamine (serotonin) receptor 1B, G protein*-*coupled*) on GGA3, *RIMBP2* (*RIMS binding protein 2*) on GGA15, *RGS3* (*regulator of G*-*protein signaling 3*) on GGA17, and *KHDRBS1* (*KH domain containing, RNA binding, signal transduction associated 1*) on GGA23.

**Table 4:** SNPs significantly associated with residual feed intake and feed conversion ratio detected in the bivariate GWAS analysis

Traits^a^	SNP	GGA^b^	Position	*P* value	MAF^c^	β^d^		Candidate/nearest gene	Location (kb)^e^
						T1	T2		
RFI	rs312274422	3	79,252,286	3.77e−10	0.28 (G/T)	−0.24	10.06	HTR1B	U 239.93
	rs315604099	15	3,307,271	5.29e−7	0.27 (A/G)	−0.08	9.01	RIMBP2	Intron 15
	rs312873273	17	1,366,852	1.44e−11	0.21 (G/C)	−1.90	10.49	RGS3	Intron 14
	rs315868235	23	5,055,731	1.62e−7	0.03 (C/T)	−0.46	−23.68	TMEM39B	U 0.34
FCR	rs14841309	1	68,353,988	3.57e−7	0.40 (G/C)	−0.01	−0.21	PPFIBP1	U 3.10
	rs13880743	1	66,797,959	4.40e−7	0.46 (G/A)	0.09	−0.13	ST8SIA1	Intron 3
	rs15303811	1	67,832,808	4.92e−7	0.45 (T/C)	0.03	−0.18	ITPR2	Intron 54
	rs317525285	1	66,971,254	6.18e−7	0.39 (A/T)	0.05	−0.17	C3AR1	D 27.52
	rs13882373	1	68,400,729	6.26e−7	0.46 (A/T)	−0.11	0.11	PPFIBP1	Intron 4
	rs314631602	1	68,131,791	6.67e−7	0.44 (A/T)	0.07	−0.15	C1H12orf11	Intron 2
	rs317881125	1	67,475,629	6.82e−7	0.17 (T/A)	−0.16	0.14	LOC418209	D 34.44
	rs315936100	1	67,956,079	7.74e−7	0.50 (A/G)	−0.11	0.11	ITPR2	Intron 27

^a^RFI and FCR represent residual feed intake and feed conversion ratio, respectively

^b^Chicken chromosome

^c^Allele frequency of the first listed marker

^d^Effect of allele substitution; T1 and T2 represent laying periods between 37 and 40 weeks and between 57 and 60 weeks, respectively

^e^U and D indicate that the SNP is upstream and downstream of a gene, respectively

### Feed conversion ratio

Univariate GWAS analysis identified 17 SNPs that were associated with FCR (*P* value < 1.69e−5), including six SNPs for FCR1 and 11 SNPs for FCR2. Together, the six SNPs for FCR1 explained 3.00 % (SE = 0.02) of its phenotypic variance. Five of these six SNPs were on GGA7 in a 1.94 Mb region between 15.86 and 17.80 Mb, and three of them, i.e. *rs317101341*, *rs317398212,* and *rs313786079* were in a haplotype block that spanned 324 kb (see Additional file 4: Figure S2). The most significant association was found between FCR1 and SNP *rs317101341* (*P* value = 2.59e−6), which was located at 42.52 kb upstream of the *CDCA7* (*cell division cycle associated 7*) gene (Table 3). Notably, all SNPs on GGA7 had a consistently favorable effect on FCR with a low MAF (<0.1), which indicates that most of the birds in this population carried the dominant genotype. The sixth SNP associated with FCR1 was located on GGA1 at 56.00 Mb in the *TBXAS1* (*thromboxane A synthase 1 (platelet)*) gene. Together the 11 SNPs associated with FCR2 explained 5.14 % (SE = 0.03) of the phenotypic variance. The detailed annotations of these SNPs are in Additional file 3: Table S5, among which SNP *rs312620976* with the strongest association signal for FCR2 (*P* value = 1.86e−6) was situated 16.04 kb downstream of the *CETP* (*cholesteryl ester transfer protein*) gene on GGA11 (Table 3).

**Fig. 4:**
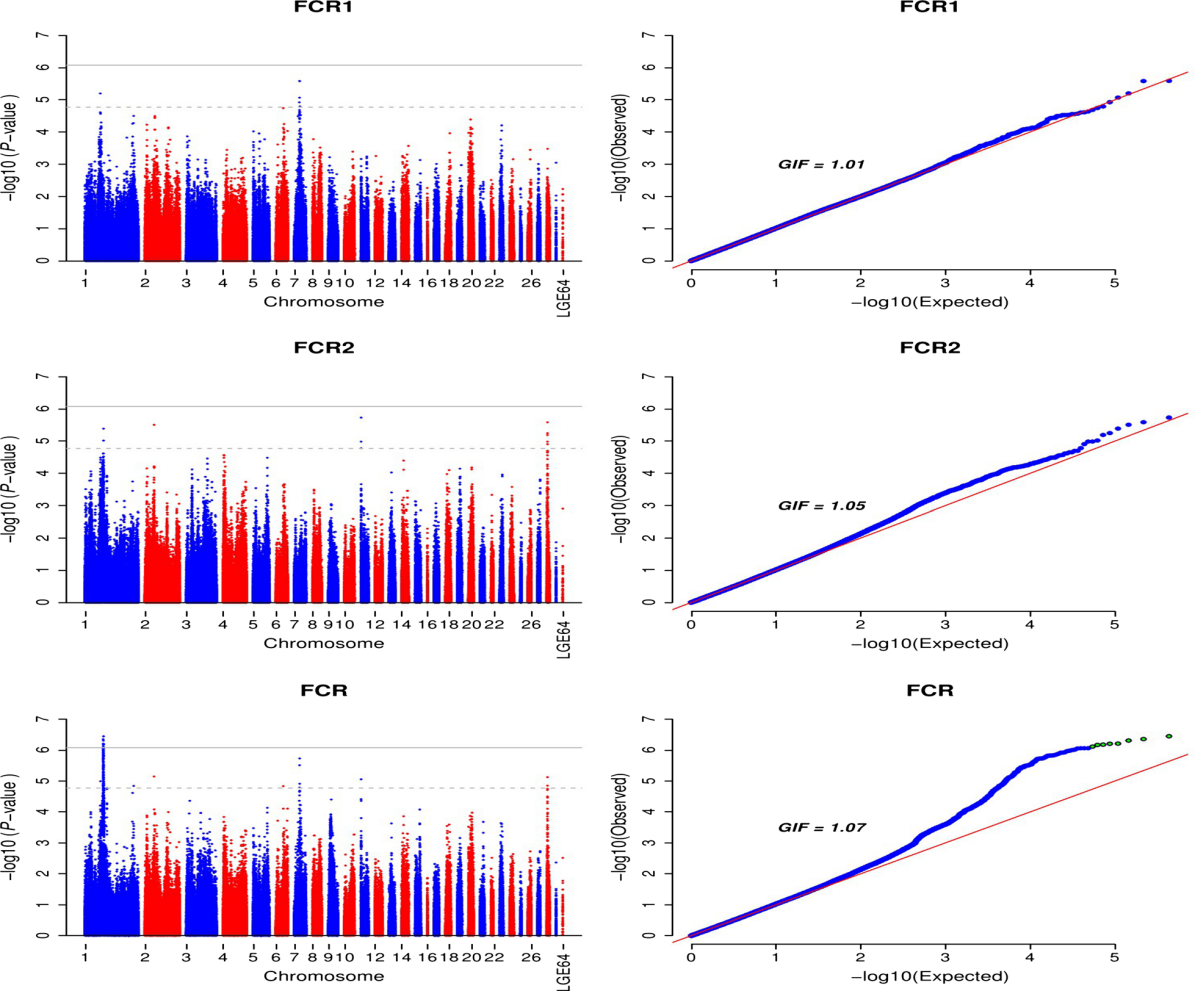
Manhattan and Q–Q plots of genome-wide association *P* values for feed conversion ratio. *FCR1* and *FCR2* represent feed conversion ratio in laying periods between 37 and 40 weeks and between 57 and 60 weeks, respectively; *FCR* represents feed conversion ratio in the bivariate analysis; *LGE64* linkage group LGE64, *GIF* genomic inflation factor. The *horizontal gray* and *gray dashed lines* indicate the whole-genome significance (*P* value = 8.43e−7) and genome-wise suggestive significance thresholds (*P* value = 1.69e−5), respectively

The bivariate analysis identified a significantly associated genomic region that contained eight SNPs on GGA1 between 66.80 and 68.40 Mb for FCR (Table 4; Fig. 4). These SNPs were in high LD and located in two haplotype blocks that spanned 480 and 268 kb, respectively (see Additional file 5: Figure S3). In this region, it is very likely that one or two putative QTL have similar effects on FCR in each of the two laying periods, which is consistent with the high estimates found for the SNP-based genetic correlations (Table 1). These eight SNPs explained 1.96 and 1.45 % of the phenotypic variance for FCR1 and FCR2, respectively. The most significant SNP *rs14841309* in haplotype block 2 was located 3.10 kb upstream of the *PPFIBP1* gene. This haplotype block also contained the gene *ITPR2* (*inositol 1,4,5*-*trisphosphate receptor, type 2*) and an uncharacterized gene *LOC418209*.

## Discussion

GWAS is a powerful tool for the genetic analysis of important production traits in farm animals. Recently, GWAS have identified a number of QTL, candidate regions, and SNPs associated with daily FI, RFI, and FCR in beef cattle [27, 28] and swine [29–31]. To our knowledge, the work presented here is the first GWAS of feed intake and efficiency during the laying period in chickens.

### GWAS analysis

Similar to previous findings in dairy cattle [32], the bivariate analysis detected more associations than the univariate analysis, including genetic variants that affected the same trait expressed in each of the laying periods and genetic variants that affected only expression of a trait in one of the laying periods. These results confirm that multivariate analysis increases the power of detection when genetic correlations occur between traits [32–34].

In practice, it is usually considered that a genomic control inflation factor λ less than 1.05 indicates no population stratification [16]. In the present study, slight population stratification was found in the univariate and bivariate GWAS analyses for FI. Generally, genomic inflation is caused by family structure, cryptic relatedness, and genotyping errors, which lead to spurious associations. However, the powerful mixed model approach that we used here can account for the genomic inflation effectively [16]. Moreover, no strong evidence of population stratification was found for RFI and FCR, for which a limited number of significant genetic variants were detected, which indirectly indicates the polygenic inheritance of FI. Thus, we attributed the observed inflation in our study to the presence of polygenic inheritance [35], since higher λ are obtained when the number of significantly associated SNPs is large. Furthermore, after conditional analysis, the corresponding associated signals disappeared and λ decreased, which demonstrated that genomic inflation was caused by large number of associated SNPs.

### Associations with feed intake

The region associated with FI1 on GGA4 was concordant with a previously reported QTL for a layer population [36]. Interestingly, the five SNPs that were detected by conditional analysis were located within the *NCAPG*-*LCORL* (*non*-*SMC condensin I complex, subunit G, and ligand dependent nuclear receptor corepressor*-*like*) locus. This locus is associated with stature in humans, cattle, and horses [37, 38] and is significantly associated with FI in beef cattle [39]. Based on this evidence, it is likely that this locus also affects FI in chickens but further validation on a larger population is necessary. For FI2, the most significant SNP *rs316723909* was situated upstream of the *GIP* (*gastric inhibitory polypeptide*) gene, which encodes an incretin hormone inducing insulin secretion [40] and has a role in mediating appetite and energy intake [41].

The region that we detected on GGA1 for FI was located downstream of a previously reported QTL [42]. The most significant SNP associated with FI1 was within the *CAB39L* (*calcium binding protein 39*-*like*) gene. The protein CAB39L, also known as MO25beta encoding calcium-binding protein 39-like, is expressed in the nervous system and sensory organs in mice [43]. It activates and interacts with the serine/threonine kinase 11/liver kinase (B1STK11/LKB1), which catalyzes the process of phosphorylation to activate AMP-activated protein kinase (AMPK), thus plays a role in the regulation of food intake [44, 45]. Moreover, studies in chickens have suggested that an AMPK pathway similar to that in mammals exists in chickens [46]. Therefore, *CAB39L* may be a promising candidate gene for FI1, and further functional approaches are necessary to validate this hypothesis. Moreover, the eight significant SNPs that were identified for both FI1 and FI2 on GGA1 had a consistently positive effect, which indicates that this candidate region has a similar effect on daily feed intake in each period and is responsible for the high genetic correlation for feed intake between the two laying periods [7, 47, 48].

### Associations with residual feed intake

RFI is a complex trait that is affected by many factors including feed-, growth- and egg-related traits. The changes observed in these traits between the two laying periods lead to different associated genetic variants between RFI1 and RFI2. In addition, environmental conditions can differ between the two laying periods and impact the genetic effect of these variants [49]. This might explain why the identified variants differed between RFI1 and RFI2, and significant SNPs were detected only for RFI2. The region on GGA27 that is associated with RFI2 can be considered as a novel candidate region, since no QTL or SNP was previously reported in this region. The highly significant SNP is located in a novel gene *LOC429785* that has no known function in chickens. In humans, *LOC429785* is a member of the GIRK (G protein-coupled inwardly-rectifying potassium channel) family and mediates several functions in the central nervous system [50]. Onteru et al. [31] reported that the *GNG4* (*guanine nucleotide binding protein 4*) gene was associated with RFI in pigs, which acted by activating inward rectifier potassium channels, a mechanism that needs to be further studied in chickens. In the present study, the most significant SNP for RFI1 was in a region for which no gene information was available. The closest annotated gene to this SNP was *carnitine palmitoyltransferase 1A* (*CPT1A*) located 21.57 kb downstream. CPT1 is inhibited by malonyl-coenzyme A, which is involved in the hypothalamic regulation of energy expenditure and food intake [51–53]. Ka et al. [54] demonstrated differential expression of *CPT1B* in two chicken lines that were selected for high (HWS) and low (LWS) body weight; furthermore, higher levels in the hypothalamus but lower levels in muscle in the HWS compared with that in the LWS line were correlated with increased appetite and food intake.

Unlike the results of the bivariate analysis for FI, the SNPs that were significantly associated with RFI2 on GGA27 did not reach genome-wide significance in the bivariate analysis for RFI. This may be due to a discrepancy between the effects of SNPs on FI and RFI, i.e., the phenotypic variance explained by the SNPs associated with FI on GGA1 was much larger than that explained by the SNPs associated with RFI on GGA27. Four highly significant SNPs were found to be associated with RFI in the bivariate analysis, of which two, *rs312873273* and *rs312274422*, were located at 79.25 Mb on GGA3 and at 1.37 Mb on GGA17, which corresponds to two interesting candidate genes, *HTR1B* and *RGS3*, respectively. The HTR1B protein is a G-protein-linked receptor for serotonin [55] and is associated with eating behavior and appetite control [56, 57]. RGS3 interacts directly with G-proteins and some evidence suggests that RGS family members may indirectly affect proteins in the MAPK (mitogen-activated protein kinase) signal transduction pathways [58]. Furthermore, it has been suggested that MAPK is associated with residual feed intake in pigs and beef cattle [31, 59]. Further research is needed on these two candidate genes in chickens.

### Associations with feed conversion ratio

In the univariate GWAS analysis, no SNP reached the genome-wide significance level for FCR1 and FCR2, which indicated that FCR was a complex trait controlled by several or numerous genes, each with a small effect. Thus, a larger sample size is needed to detect associations with FCR. In addition, applying a looser statistical significance threshold by using a false discovery rate method or a Bayesian model may identify more interesting candidate genes. Nevertheless, based on genome information for the significant SNPs, we were able to suggest several promising candidate genes. For FCR2, the most significant SNP *rs312620976* is within a promising candidate gene *CETP*, which is involved in the transfer of cholesteryl ester from high-density lipoproteins to other lipoproteins [60]. Previously, Sato et al. reported that impaired CETP activity played an important role in egg production of laying hens [61], and egg production is one of important components affecting FCR [9]. Moreover, *ITPR2*, a promising candidate gene that was identified in the bivariate analysis, encodes a receptor for inositol triphosphate and a calcium channel [62]. Since the inositol trisphosphate receptor type 2 is involved in ion transfer in the hen’s uterus [63], it is possible that this gene will impact FCR by affecting egg formation.

## Conclusions

This study identified eight genomic regions that are significantly associated with FI. Seven of these associations are reported for the first time, which demonstrates the power of high-density SNP arrays and distinct parental lines. One of the significant allele substitution effects was observed for a SNP associated with RFI in the laying period between 57 and 60 weeks and caused a 3.35 g/day phenotypic difference. It is located in *LOC429785*, and is a candidate gene that possibly exerts a potential function in the control of RFI. Furthermore, we identified the *CETP* and *ITPR2* genes that influenced FCR by affecting egg production. Our results provide valuable knowledge on SNP and candidate genes that are involved in the genetic architecture of FI, RFI, and FCR in two laying periods of chickens. The SNP or regions that are associated with feed intake and efficiency can be used as fundamental information in marker-assisted or genomic selection.

## Additional files

10.1186/s12711-015-0161-1 Multivariate linear model for the calculation of RFI in two laying periods.
10.1186/s12711-015-0161-1 Manhattan and Q–Q plots obtained from conditional GWAS analysis for daily feed intake. FI1 and FI2 represent daily feed intake in laying periods between 37 and 40 weeks and between 57 and 60 weeks, respectively and FI is daily feed intake from the bivariate analysis; GIF = genomic inflation factor. The horizontal gray = and gray dashed lines indicate the whole-genome significance (*P* value = 8.43e−7) and genome-wise suggestive significance thresholds (*P* value = 1.69e−5), respectively.
10.1186/s12711-015-0161-1 Detailed information on SNPs associated with feed intake and efficiency that were detected by the univariate and bivariate GWAS analyses. **Table S3.** Detailed information on SNPs associated with feed intake and efficiency that were detected by conditional GWAS analysis. **Table S4.** SNPs associated with residual feed intake in the two laying periods (*P* value < 1.69e−5). **Table S5.** SNPs associated with feed conversion ratio in the two laying periods (*P* value < 1.69e−5).
10.1186/s12711-015-0161-1 Haplotype blocks on GGA7 for feed conversion ratio. Linkage disequilibrium plot for the SNPs on GGA7 showing association (*P* value < 1.69e−5) with feed conversion ratio in the laying period between 37 and 40 weeks. Solid lines mark the identified blocks.
10.1186/s12711-015-0161-1 Haplotype blocks on GGA1 for feed conversion ratio. Linkage disequilibrium plot for the SNPs on GGA1 showing significant association (*P* value < 8.43e−7) with feed conversion ratio in the bivariate analysis. Solid lines mark the identified blocks.
