# Supplementary material for: Genome-wide association studies for feed intake and efficiency in two laying periods of chickens
Source: Genet Sel Evol. 2015 Oct 16;47:82. doi: 10.1186/s12711-015-0161-1 (PMC4608132; doi:10.1186/s12711-015-0161-1)
Supplement: Supplementary file 1 — 10.1186/s12711-015-0161-1 Multivariate linear model for the calculation of RFI in two laying periods. [file 12711_2015_161_MOESM1_ESM.docx]

**Laying period from 37 to 40 weeks of age**

| **1,856 hens**  Residuals:  Min 1Q Median 3Q Max  -34.65 -4.49 0.21 4.73 29.12  Coefficients:  Estimate Std. Error t value Pr(>\|t\|)  (Intercept) 55.84 1.48 37.70 < 2e-16 ***  EM 0.47 0.02 29.10 < 2e-16 ***  MBW 0.01 0.001 12.36 < 2e-16 ***  BWG 0.63 0.08 7.71 2.05e-14 ***  Residual standard error: 7.148 on 1852 degrees of freedom  Multiple R-squared: 0.38, Adjusted R-squared: 0.38  F-statistic: 375.2 on 3 and 1852 DF, p-value: < 2.2e-16 | **1,534 hens**  Residuals:  Min 1Q Median 3Q Max  -24.60 -4.37 0.28 4.64 22.68  Coefficients:  Estimate Std. Error t value Pr(>\|t\|)  (Intercept) 46.83 2.05 22.87 < 2e-16 ***  EM 0.48 0.03 18.03 < 2e-16 ***  MBW 0.13 0.01 12.98 < 2e-16 ***  BWG 0.50 0.09 5.58 2.89e-08 ***  Residual standard error: 6.576 on 1530 degrees of freedom  Multiple R-squared: 0.32, Adjusted R-squared: 0.31  F-statistic: 234.8 on 3 and 1530 DF, p-value: < 2.2e-16 |
| --- | --- |

**Laying period from 57 to 60 weeks of age**

| **1,802 hens**  Residuals:  Min 1Q Median 3Q Max  -46.07 -4.53 0.31 5.37 26.32  Coefficients:  Estimate Std. Error t value Pr(>\|t\|)  (Intercept) 68.76 2.08 33.09 < 2e-16 ***  EM 0.35 0.01 23.60 < 2e-16 ***  MBW 0.11 0.01 11.77 < 2e-16 ***  BWG 0.49 0.06 7.63 3.72e-14 ***  Residual standard error: 8.14 on 1798 degrees of freedom  Multiple R-squared: 0.30, Adjusted R-squared: 0.30  F-statistic: 256.6 on 3 and 1798 DF, p-value: < 2.2e-16 | **1,534 hens**  Residuals:  Min 1Q Median 3Q Max  -34.80 -4.33 0.24 5.00 19.36  Coefficients:  Estimate Std. Error t value Pr(>\|t\|)  (Intercept) 69.80 2.05 34.07 < 2e-16 ***  EM 0.29 0.02 13.66 < 2e-16 ***  MBW 0.11 0.01 12.66 < 2e-16 ***  BWG 0.30 0.07 4.52 6.81e-06 ***  Residual standard error: 7.092 on 1530 degrees of freedom  Multiple R-squared: 0.21, Adjusted R-squared: 0.21  F-statistic: 139.2 on 3 and 1530 DF, p-value: < 2.2e-16 |
| --- | --- |
