# Supplementary figures and images for: Genome-wide association studies for feed intake and efficiency in two laying periods of chickens
Source: Genet Sel Evol. 2015 Oct 16;47:82. doi: 10.1186/s12711-015-0161-1 (PMC4608132; doi:10.1186/s12711-015-0161-1)

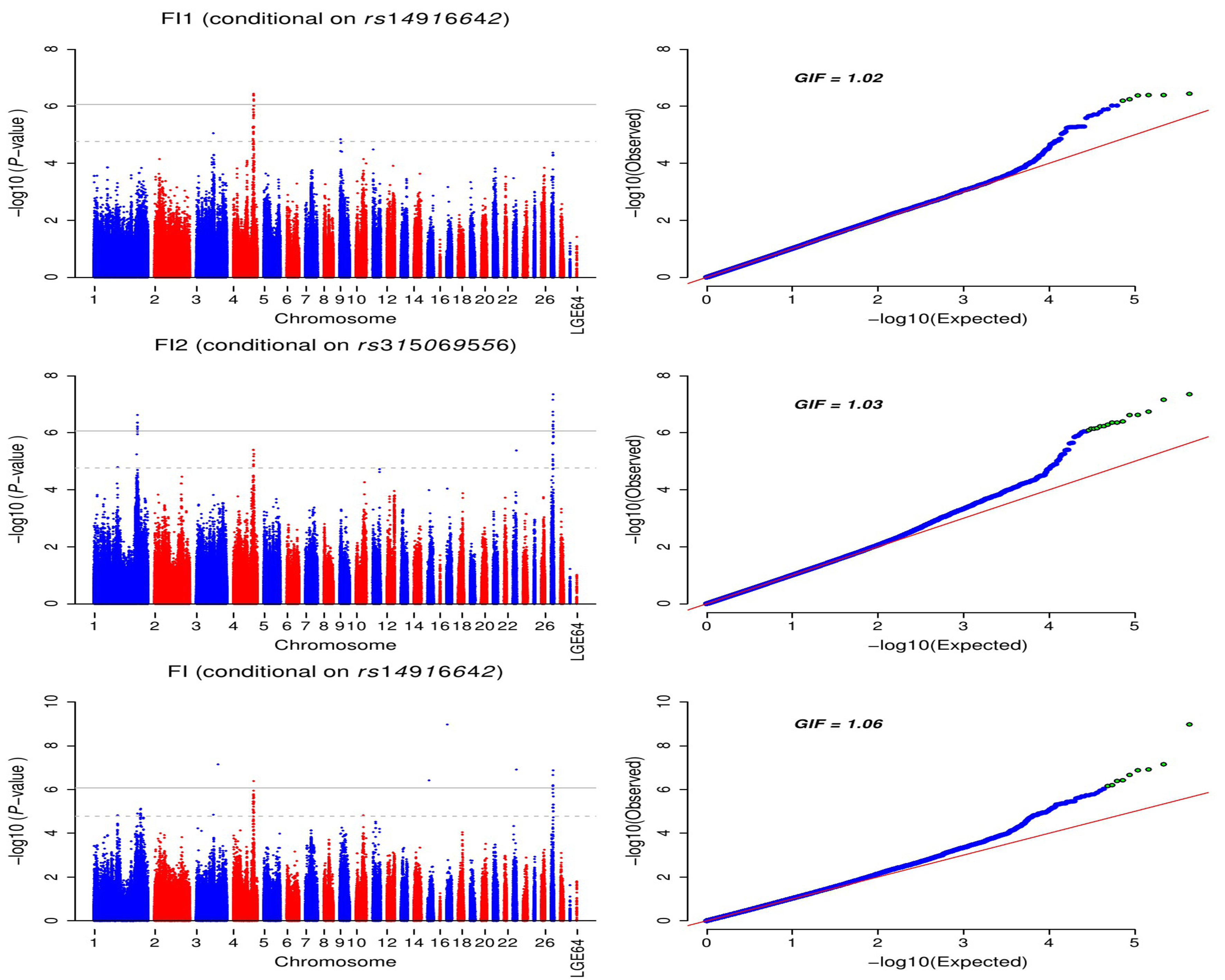

Supplement: Supplementary file 2 — 10.1186/s12711-015-0161-1 Manhattan and Q–Q plots obtained from conditional GWAS analysis for daily feed intake. FI1 and FI2 represent daily feed intake in laying periods between 37 and 40 weeks and between 57 and 60 weeks, respectively and FI is daily feed intake from the bivariate analysis; GIF = genomic inflation factor. The horizontal gray = and gray dashed lines indicate the whole-genome significance (P value = 8.43e−7) and genome-wise suggestive significance thresholds (P value = 1.69e−5), respectively. [file 12711_2015_161_MOESM2_ESM.tiff]

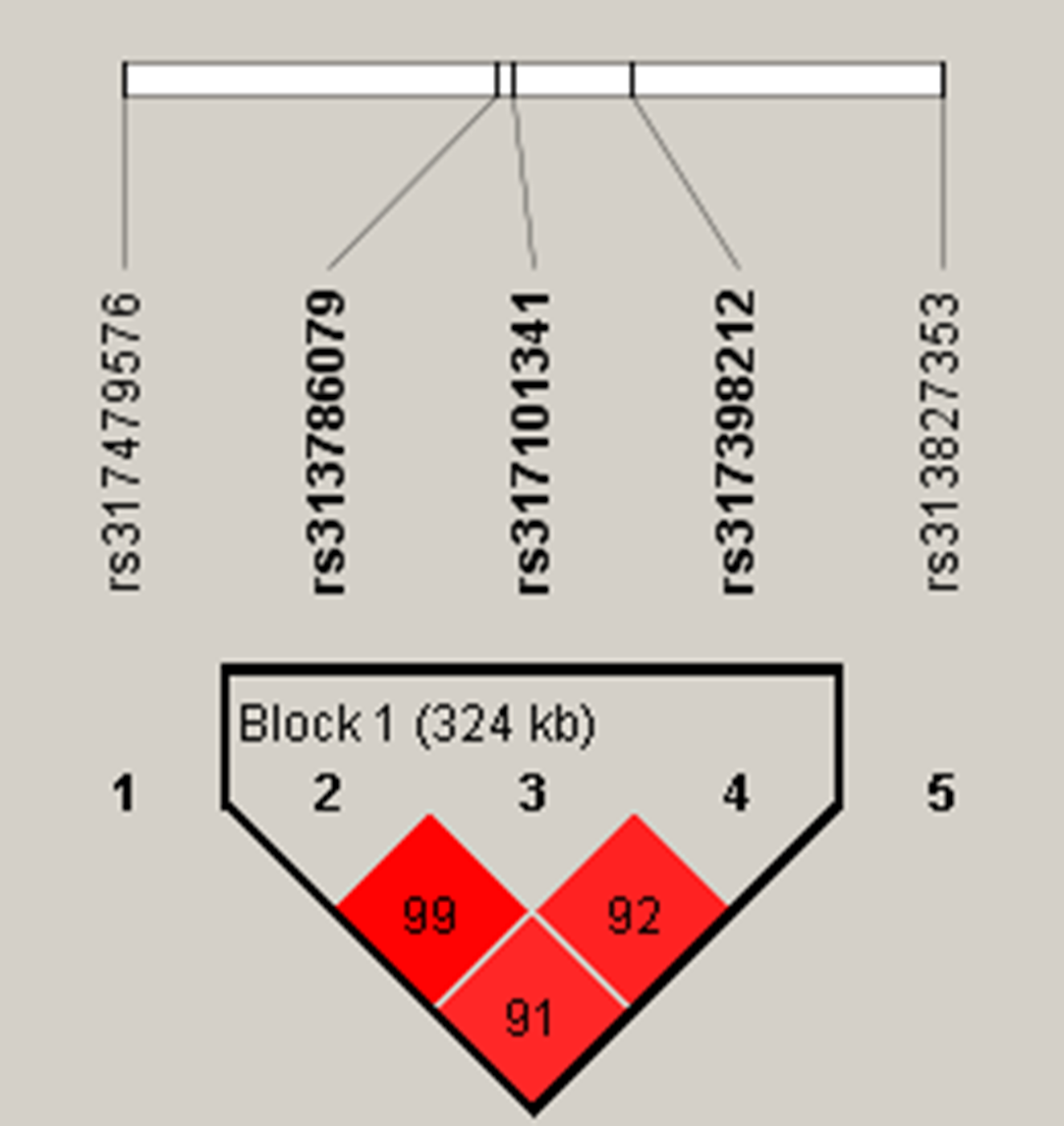

Supplement: Supplementary file 4 — 10.1186/s12711-015-0161-1 Haplotype blocks on GGA7 for feed conversion ratio. Linkage disequilibrium plot for the SNPs on GGA7 showing association (P value < 1.69e−5) with feed conversion ratio in the laying period between 37 and 40 weeks. Solid lines mark the identified blocks. [file 12711_2015_161_MOESM4_ESM.tiff]

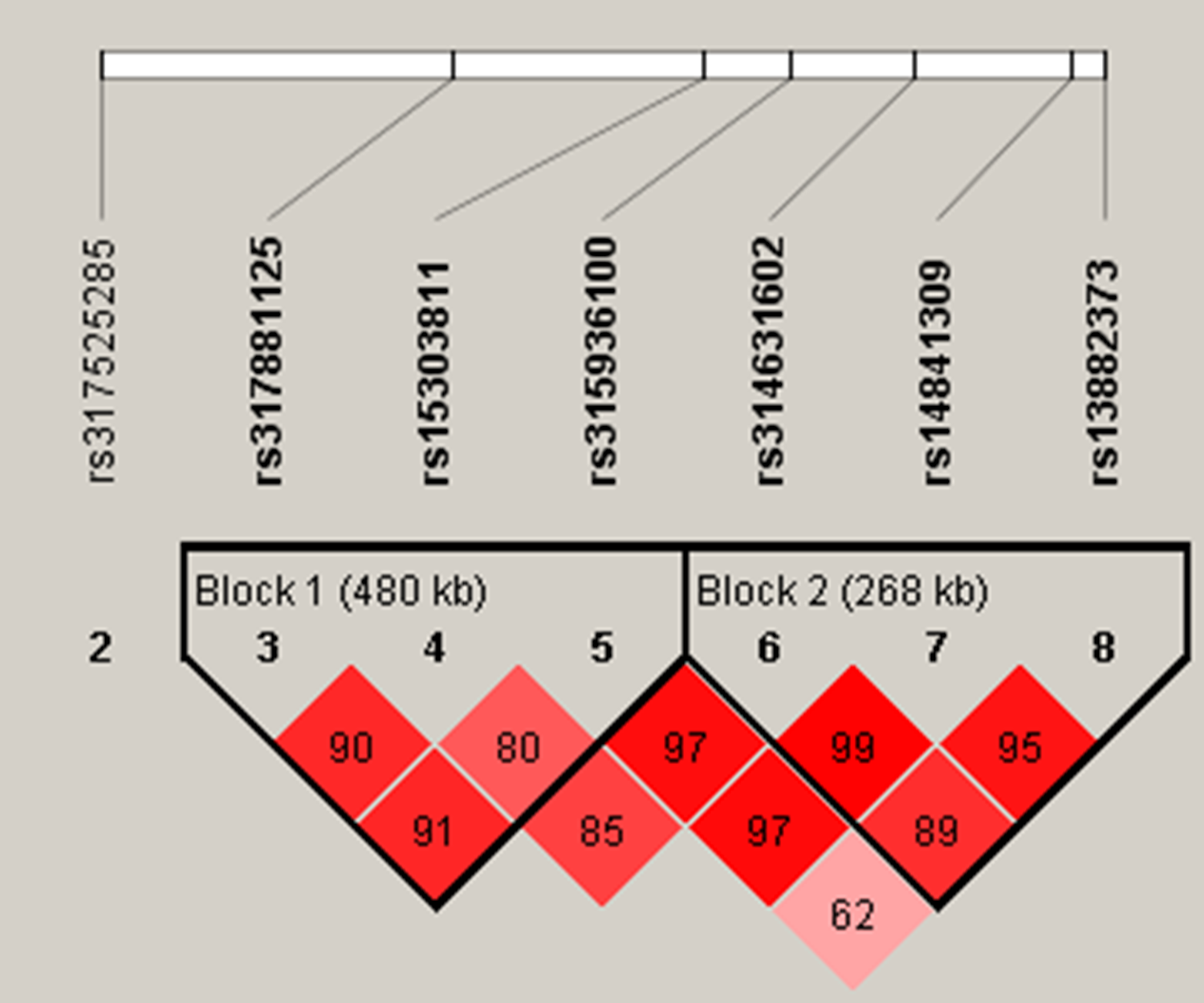

Supplement: Supplementary file 5 — 10.1186/s12711-015-0161-1 Haplotype blocks on GGA1 for feed conversion ratio. Linkage disequilibrium plot for the SNPs on GGA1 showing significant association (P value < 8.43e−7) with feed conversion ratio in the bivariate analysis. Solid lines mark the identified blocks. [file 12711_2015_161_MOESM5_ESM.tiff]
